# Syrian refugee women’s access to family planning services and modern contraception during overlapping crises in Bekaa, Lebanon

**DOI:** 10.1186/s12905-023-02613-8

**Published:** 2023-09-06

**Authors:** Rima Mourtada, Andrea J. Melnikas

**Affiliations:** Population Council. One Dag Hammarskjold Plaza, New York, NY 10017 USA

**Keywords:** Syrian refugees, Covid-19, Family planning, Contraception, Reproductive health services

## Abstract

**Background:**

Political, financial, and pandemic crises in Lebanon have affected both provision of reproductive health services including family planning and modern contraception methods as well as women’s interest and ability to seek those services. This study aims to explore the impact of the compounding crises on the provision and use of family planning services including modern contraception methods for Syrian refugees in Lebanon focusing on the perspectives of Syrian refugee women.

**Methods:**

We carried out 12 Focus Group Discussions (FGDs) with 119 Syrian refugee women recruited from two cities in West Bekaa, Lebanon from inside and outside the informal tented settlements. We used Skype video calls to moderate the FGDs due to the limited mobility at the time of the study because of Covid-19. We used thematic analyses to analyse the data.

**Results:**

The crises seemed to exacerbate supply side barriers, which influenced provision of family planning services and women’s demand for them. These included Covid-19 regulations and maltreatment by staff at public health facilities, disruption of outreach reproductive health services that provide family planning and modern contraception, and reduced supply of modern contraception methods. On the demand side, women reported financial limitations in accessing and paying for services, concern over being infected with Covid-19, and concerns about insecurity.

**Conclusions:**

We suggest possible interventions to address these challenges and better reach these populations. These include using mobile health technology (mHealth) that may provide contraceptive counselling and/or can inform refugee women about where they may receive family planning and modern contraception. These services may also support Syrian refugees to access care they are entitled to receive and may also address disruptions in service provision due to overlapping crises, including availability and rising costs of contraceptives. These can be coupled with mobile outreach reproductive health services that provide family planning. We also suggest considering the provision of Long Acting Reversible Contraception (LARC) for Syrian refugee women, which would reduce a barrier of needing to revisit health facilities to obtain an additional supply of contraception pills.

**Supplementary Information:**

The online version contains supplementary material available at 10.1186/s12905-023-02613-8.

## Background

Before the conflict erupted in Syria in 2011, the majority of women had easy access to reproductive health (RH) services [1]. In 2009, 88% of Syrian women had at least one antenatal care visit with a skilled provider and 83.6% of demand for family planning (FP) was met [2].

Since the Syrian conflict, Lebanon has been hosting the largest number of Syrian refugees compared to other neighbouring countries. Currently, there are 805,326 registered Syrian refugees in Lebanon [3]: 39% are in Bekaa, 28% are in North Lebanon, 22% are in Beirut and 11% are in South Lebanon [3]. The deteriorating living conditions due to displacement influenced all areas of refugees’ lives including their access to and use of RH services [1].

**Fig. 1:**
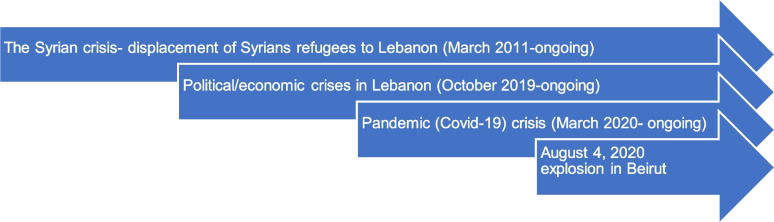
Timeline of the compounding crises in Lebanon

Lebanon has been dealing with consecutive and overlapping crises since October 2019 (Fig. 1) that caused severe deterioration of the living conditions for Lebanese nationals and Syrian refugees. A sudden stop in capital inflows in October 2019 resulted in a financial crisis affecting the exchange rate and causing inflation [4]. Given the financial crisis, the Lebanese government decided to impose a new tax on WhatsApp voice calls, which led to political turmoil that was manifested by protests and road blocks across the country [5]. The financial and political crises were soon aggravated by the Lebanese government’s measures to control Covid-19 transmission, which included closing all borders, shuttering public and private institutions, and two national lockdowns between March 2020 and August 2021 [4].

These crises were soon followed by the August 4, 2020 port explosion in Beirut that resulted in 220 deaths, 7000 injuries and the destruction of 300,000 homes in the capital [6], which exacerbated the financial crisis and increased Covid-19 transmission. Additionally, the destruction of many hospitals due to the explosion strained the health system and weakened its ability to deal with the increasing number of Covid-19 cases. Therefore, the Lebanese government imposed additional national lockdown measures.

The combined effects of the consecutive overlapping crises, which were also exacerbated by the repercussions of the explosion, affected all areas in the lives of people in Lebanon including Syrian refugees. The Lebanese Pound (LBP) was devalued by 80% and inflation exceeded 140% [7]. The proportion of Syrian refugees who were living below the Survival Minimum Expenditure Basket, the absolute minimum amount required to cover lifesaving needs, increased from 55% in 2019 to 88% in 2021. Half of Syrian households suffered from food insecurity in 2021 [8]. Access to health care was severely influenced by the crises and was mainly impeded by financial limitations [8].

The current challenges in Lebanon are likely to contribute further to limiting access to RH services and FP by affecting supply side determinants (features related to the health system that impede service uptake by individuals, households or the community) and demand side determinants (factors that influence the ability to use health services at individual, household or community level) [9].

The disruption of the supply chain of contraceptive products caused by Covid-19 pandemic at the global level [10], a health system strained by the 4^th^ of August explosion, as well as the local government’s effort to limit the transmission of Covid-19 at the country level, likely resulted in a reduction of the provision of what is considered as “less essential” services such as provision of FP services [10].

With regard to the demand side, the challenges arising from Covid-19 national lockdown, starting March 2020, were aggravated by the existing political and financial crises, which caused an additional decline in job opportunities for Syrian refugees [8]. Inflation and bank restrictions impeded the cash assistance process provided by the United Nations (UN) [8]. Moreover, the cash assistance received was no longer sufficient due to the rising prices and the unofficial market exchange rate that devalued the LBP [8]. The curfews inflicted by many local municipalities in Bekaa to contain Covid-19 transmission [11], in addition to the refugees’ fear of being infected have likely impacted the refugees’ mobility and decreased their access to basic needs including health services. Therefore, it is expected that access to FP services and modern contraception have decreased further since the crises.

Frequently, women and girls are disproportionately affected during conflict and displacement as disruption in access to RH services can lead to poor sexual and reproductive health outcomes [12]. Provision of health care for Syrian refugees is based on a collaborative efforts between the Ministry of Public Health (MoPH), the United Nations High Commissioner for Refugees (UNHCR), and local and international Non-Governmental Organizations (NGOs) and humanitarian aid agencies [13]. Typically, refugees can access antenatal care, postnatal care, and FP (including the provision of contraception) at the MoPH primary health care (PHC) facilities [13]. UNHCR helps refugees access PHC services by largely subsidizing the cost of consultations and medication through 158 PHC facilities [14, 15]. Also, there are 25 mobile medical units operated by NGOs that provide free consultations and medication including modern contraception when refugees cannot access PHC facilities [13]. Syrian refugee women can also receive RH care including FP at private clinics and hospitals and can access certain contraception types such as birth control pills and condoms at pharmacies. However, provision of RH services to refugees before the crises was not optimal due to the continuous influx of Syrian refugees, which seemed to burden the existing health structures [16].

Barriers to RH services including FP among Syrian refugees in Lebanon are not a recent occurrence: there was an unmet need for FP post displacement [1]. The barriers that Syrian refugee women faced when accessing RH services and modern contraception in Lebanon included maltreatment by health providers, long waiting times at PHC facilities, cost of modern contraception at private health facilities, limited mobility due to cultural reasons as well as lack of transportation, limited awareness about the discounted provision of RH services and modern contraception at PHC facilities, lack of appropriate documentation required to obtain modern contraception provided at some facilities/organizations and provision of contraception methods that did not meet the needs of participants [17–22]. Studies documenting those barriers were carried out before those multiple crises, when service provision was relatively better and when Syrian refugees had better mobility, livelihoods, and better access to services. A recent systematic review of the indirect impacts of Covid-19 on Sexual and Reproductive Health (SRH) emphasized the research gaps in understanding how Covid-19 disruptions in SRH services provision, access and/or utilization have impacted underserved populations [23].

The decrease in the supply of and the demand for FP services will likely increase the number of unintended pregnancies. Recent analyses (from 2020) predict that a 10% drop in the use of short and long-acting reversible contraceptives may lead to 15,401,000 additional unintended pregnancies in low and middle-income countries (LMIC) [10]. The implications of such estimates are serious in a population where the rates of child marriage and early pregnancy are likely to be high [24, 25]. Even when the national lockdown is over, the financial and political crises are unlikely to improve, and the situation of Syrian refugees is expected to worsen.

This study contributes to the current knowledge on FP access among displaced populations by exploring the most recent supply and demand side barriers that Syrian refugees face when accessing FP services. What makes this study important and different from other studies that explore the effects of Covid-19 on access to FP services is that Covid-19 in Lebanon was happening among other overlapping crises, which amplified the disruption of access to such services. Findings will have important implications for improving access to FP services by suggesting some strategies and potential delivery mechanisms/interventions that address the existing barriers and improve access to services given all of the current challenges.

## Methods

### Aims of the study

Given the recent political, financial and pandemic challenges in Lebanon, this study seeks to understand:

1. The current perspectives of Syrian refugee married women/girls regarding provision of FP services and modern contraception methods for Syrian refugees in Lebanon.
2. Use of FP services and modern contraception methods among Syrian refugee married women/girls by exploring their current attitudes and practices.

This study is part of a larger study that also explored the effect of the crises on Syrian refugees’ living conditions and fertility preferences and practices [26].

### Conceptual framework

In this study, we used an adapted conceptual framework (Additional file 1), which describes the supply and demand side barriers that impede access to RH services under each of the four main dimensions of care described by (Peters et al., 2008) [27] and (Jacobs et al., 2012) [9]: “Availability —having the right type of care available to those who need it, such as working hours and waiting times that meet demands of those seeking care, as well as having the appropriate type of service providers and materials; Geographic accessibility—the physical distance or travel time from service delivery point to the user; Financial accessibility—the relationship between the price of services (in part affected by their costs) and the willingness and ability of users to pay for those services, as well as be protected from the financial consequences of health costs; Acceptability—the match between how responsive health service providers are to the social and cultural expectations of individual users and communities”.

### Study design and setting

**Fig. 2:**
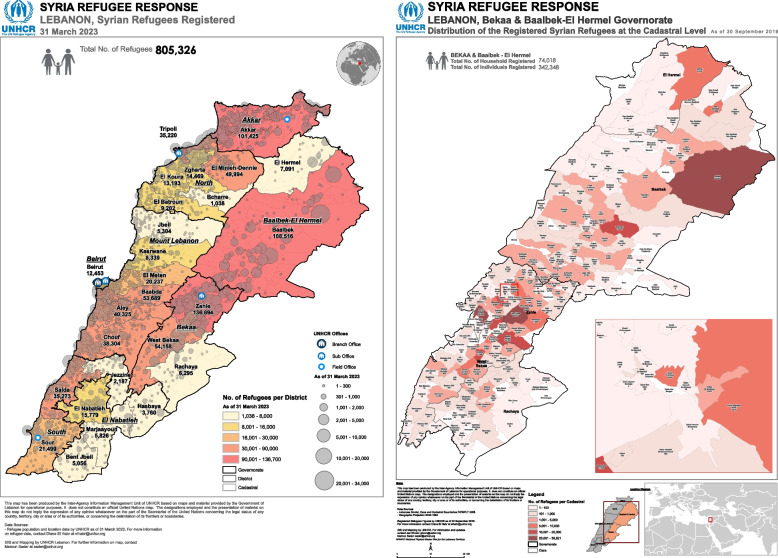
Map of Lebanon displaying study locations

This study is an exploratory qualitative study that took place in two neighbouring cities in West Bekaa that are 66 km by car from the capital Beirut, and host a large number of Syrian refugees: Bar Elias and Saadnayel (Fig. 2) [3].

Bekaa hosts 39% of the most vulnerable Syrian refugees in Lebanon [3] as many refugees live in Informal Tented Settlements (ITS) whose residents have significantly lower legal residency rates compared to those living in other shelter types [28], which will likely reduce their mobility and access to services. There are no official camps in Lebanon. Refugees live either in rented accommodation, unfinished buildings or warehouses among host community members or in ITS. Due to these severe living conditions, in addition to facing difficulties with obtaining or renewing their legal residency permit in Lebanon, Syrian refugees are generally highly mobile [8].

### Eligibility criteria

Participants in this study included the following:

a- 15–19 year old married girls. We included young girls to understand their demand for and access to FP services and modern contraception methods given the high risks of adolescent pregnancy.
b- 20–35 year old married women. We included women belonging to this age category because we wanted to understand the demand for and access to FP services and modern contraception methods for women who are at the peak of sexual activity and fertility.
c- Women who were above 35 years-old and who were mothers or mothers-in-law of girls married before age 18. We chose to include this group of women given their important role in the decision-making process regarding use of FP services and modern contraception.

### Data collection

Data collection included twelve Focus Group Discussions (FGDs). In each city (Bar Elias and Saad Nayel), we carried out:

a- Two FGDs with 15–19 year-old married girls (One FGD with girls living inside ITS and one FGD outside ITS).
b- Two FGDs with 20–35 year-old married women from inside and outside the ITS.
c- Two FGDs with women who were above 35 year-old (inside and outside the ITS) and who were mothers or mothers-in-law of girls married before age 18.

Each FGD included 9–10 women to enable us to maintain the appropriate social distancing measures to reduce the risk of Covid-19 transmission. We used FGDs because they are suitable to study complex behaviour and compare the different views shared by participants, which is not possible when using in-depth interviews [29, 30].

FGDs guides covered the following: the current hardships experienced by refugees that emerged since the political, financial, and pandemic crises; the influence of the crises on refugees’ perception of ideal number of children and their demand for modern contraception; current access to FP services and use of modern contraception; and suggestions for strategies to improve their access to FP services given the current crises. The guide was pretested in June 2020 before data collection.

### Recruitment of participants

Two Syrian female community workers who live in West Bekaa assisted with recruiting the study participants using purposive sampling from inside and outside ITS in the selected cities.

We choose one large ITS in each city to ensure the availability of the required number of eligible participants. Community workers met with eligible women in each of those settlements, explained the study and documented the contact details of those who agreed to participate.

Community workers used the help of their colleagues and social circle to connect them with eligible women who lived outside the ITS. They contacted eligible women using WhatsApp, explained the study, and documented the contact details of those who agreed to participate.

Data was collected between July and October 2020 by the first author (RM), who moderated the FGDs and who has extensive experience in qualitative research and this refugee setting. Due to the Covid-19 governmental lockdown regulations and the high risk of being infected with Covid-19 while traveling, the first author was unable to travel to Bekaa to conduct the interviews in person. Instead, FGDs were carried out by the first author using Skype video call with the help of the local community workers who were present at the research site.

FGDs with women living inside the ITS were carried out at one of the big tents inside the selected ITS and FGDs with women living outside the ITS were carried out at a community centre that was easily accessible for women who lived in both selected locations. The community workers managed the logistics, ensuring the availability of an acceptable WIFI network connection in the selected locations and ensuring no other individuals were present during the FGDs to maintain privacy. FGDs were audio recorded by one of the community workers.

### Data analyses

All interviews were conducted in Arabic, audio recorded and transcribed verbatim by the community workers. Data was analysed using thematic analysis as described by Braun and Clarke [31]. The main author translated the transcribed interviews into English. The transcripts were read several times by both authors and initial codes were generated, searching for themes, reviewing themes and finally defining, naming and organizing the final themes under demand side and supply side barriers to accessing FP services and modern contraception. The final stage involved discussing those barriers reflecting each of the four dimensions of access as shown in Additional file 1 [9, 27].

## Results

### Sample characteristics

The characteristics of the 119 women/girls who participated in FGDs are shown in Table 1.

**Table 1:** Demographic characteristics of women who participated in FGDs

*N* = 119			
	**15–19-years old (*n* = 39)**	**20–35- years old (*n* = 40)**	**Mothers and mothers-in-law (*n* = 40)**
**Mean age**	18	28	46
**Education n (%)**			
No school	4 (10)	1 (2)	9 (22)
Preparatory	21 (54)	18 (45)	16 (40)
Elementary	10 (26)	9 (23)	12 (30)
Secondary and above	4 (10)	12 (30)	3 (8)
**Age at marriage n (%)**			
12–15	25 (64)	6 (15)	11 (28)
16–17	12 (31)	15 (38)	12 (30)
18 and above	2 (5)	19 (47)	17 (42)
**Parity n (%)**			
No children	8 (21)	1 (2)	NA^a^
1	19 (49)	6 (15)	0
2–4	12 (31)	27 (68)	9 (23)
5 and above	0	6 (15)	31 (77)
**Age at first pregnancy (out of those who ever had children) n (%)**			
12–15	13 (42)	1 (3)	5 (13)
16–17	16 (52)	11 (28)	9 (22)
18–19	2 (6)	12 (31)	15 (38)
20 and above	NA	15 (38)	11 (27)

^a^NA: not applicable

### Themes

Emerging themes were organized under demand and supply side barriers that disrupted access to and use of FP services and modern contraception. The main themes are presented in Table 2.

**Table 2:** Major themes

Supply side barriers	- Covid-19 related regulations in health centres - Maltreatment by staff at health centres - Disruption of outreach RH services that provide FP and modern contraception - Limited supply of modern contraception
Demand side barriers	- Financial limitations in reaching of and paying for FP services and modern contraception - Concern over being infected with Covid-19 - Road closures and concern over insecurity

**Fig. 3:**
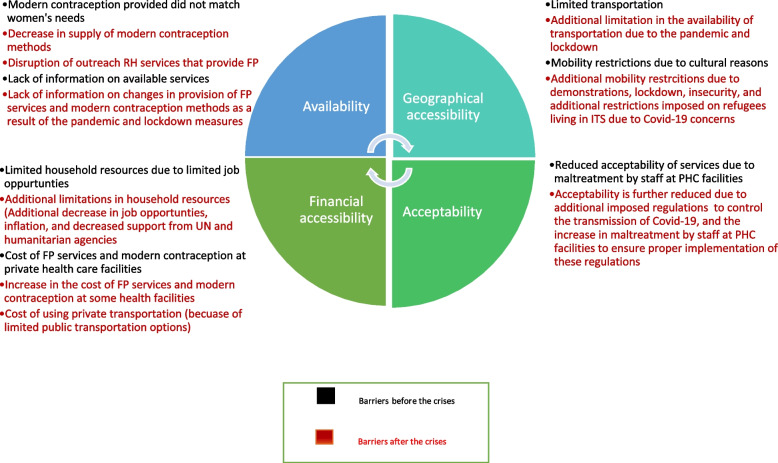
Barriers to accessing and using FP services and modern contraception before and after the crises

Despite refugees’ strong preferences to limit the number of their children as a result of the current hardships [26], the consequences of the political, financial and pandemic crises in the country seemed to impose additional barriers that restricted women’s access to FP services and modern contraception methods (Fig. 3). The supply side related barriers were Covid-19 regulations at PHC facilities, maltreatment by staff at PHC facilities, disruption of outreach RH services that provide FP and reduced supply of modern contraception methods. Demand side related barriers included financial limitations in reaching of and paying for services, concern over being infected with Covid-19 and road closures and concern over insecurity.

### Supply side barriers

#### Covid-19 related regulations in PHC facilities

Participants discussed that PHC facilities serving Syrian refugees made some changes in response to the Covid-19 pandemic, including closing the inside waiting room and requiring patients to wear masks. These changes were not always viewed favourably, as some women saw them as reducing the quality of care received and discouraged them from seeking care:

*“Although we used to wait for a long time at the health centre before the crisis, now we are concerned about having to wait outside for long hours because of Corona.” (15-19-year-old girls outside the ITS, Saad Nayel)*

However, some women reported that waiting outside did not bother them as they believed that such regulations protected their health as well. Women who reported not being dissatisfied with waiting outside were older:

*“They make us wait outside and they force us to wear a mask, and each person takes half an hour inside. It is because of Corona. They want to make sure that doctors and patients are safe. I think this is better.” (Mothers and mothers-in-law outside the ITS, Bar Elias)*

Covid-19 mitigation measures had implications on limiting the number of patients visiting the health facilities.

*“There is a lot of pressure at the nearby centre and they only agree to receive 10-15 people per day. “ (20-35-year-old women outside the ITS, Saad Nayel)*

*“Before the crisis, the doctor at the health centre used to see more patients, now they only let in 4 or 5 women so sometimes we are obliged to go to a private doctor and pay 50000 LBP.” (Mothers and mothers-in-law outside the ITS, Saad Nayel)*

#### Maltreatment by staff at health centres

Most women agreed that poor treatment by staff at PHC facilities, which was present before the crisis, has increased after the crisis, which in turn may have discouraged some women from visiting the health centre since the crisis.

*“They also need to change their hostile attitude. They became hostile because of their concern over Corona. They just want to force you out as soon as possible as if they are disgusted with you.” (20-35-year-old women inside the ITS, Bar Elias)*

Also, some women expressed their disappointment regarding the prioritization of Lebanese women over Syrian women at PHC facilities.

*“They always let Lebanese women in before Syrian women, why do they do it? Does that mean that Syrian women have Corona but Lebanese women don’t?” (20-35-year-old women outside the ITS, Saad Nayel).*

#### Disruption of outreach RH services that provide FP and modern contraception

A few women explained that they used to receive modern contraception methods from UN agencies or Non- Governmental Organizations’ (NGOs) outreach/mobile services but these services were temporarily disrupted because of Covid-19. This had important implications as access to contraception was now limited and if women still wanted to pursue contraception, they needed funds.

*“Sometimes there are outreach clinics that distribute pills. They also distribute condoms and vitamins. Sometimes they even give you an Intrauterine Device (IUD). They stopped since Corona and women need to buy them now.” (20-35-year-old women outside the ITS, Saad Nayel)*

As a result, one woman in Saad Nayel told us that she stopped taking contraception:

*“The UNHCR used to distribute pills but they stopped so I stopped taking pills a month ago.” (20-35-year-old women inside the ITS, Saad Nayel)*

#### Reduced supply of modern contraception

Women faced additional barriers in accessing modern contraception since the crises. Contraceptive pills were still being provided free of charge at many PHC facilities, however, women were given only one envelope of pills at a time instead of the whole box, which obliged them to return each month to obtain additional supply.

*“My friend needed to use pills and they only gave her one envelope at the centre instead of giving her the box that has three envelopes. “ (20-35-year-old women outside the ITS, Bar Elias)*

Returning regularly to obtain additional supplies of pills was not only inconvenient but was also embarrassing to some women.

*“One thing is bothering me is that they only give you pills for one month at the health centre, it is embarrassing for me to go and ask for them each month, I am not going to sell the pills, I don’t understand why they don’t give me more than one envelope. Instead of making things easier, they are making them harder.”(20-35-year-old women living outside the ITS, Bar Elias)*

Another woman from the same group who received her care from Doctors without Borders, reported a similar concern.

*“Now, Doctors without Borders give you the pills every two months, it is also difficult and embarrassing to ask for them every two months” .(20-35-year-old women living outside the ITS, Bar Elias)*

### Demand side barriers

#### Financial limitations in reaching of and paying for FP services and modern contraception

Most women raised concerns regarding the costs associated with accessing FP health services and/or when trying to obtain a modern contraception method, especially given their deteriorating living conditions.

Most of the women preferred to go to a private doctor before the crisis but it seems that many were no longer able to afford the private doctor’s fee.

“*We stopped going to a private doctor, we go to the health centre because it is cheaper.” (15-19-year-old girls inside the ITS, Bar Elias)*

However, PHC facilities are not always close to where they lived, which increased transportation costs and this created a financial barrier.

*“We need to pay for transportation because the health centre is not close to where we live and we do not have a car.” (15-19-year-old girls outside the ITS, Saad Nayel).*

Using overcrowded public transportation was not safe given the increased risk of Covid-19 transmission, therefore, some women used private taxis, which did not always charge consistent fees.

*“Also, if you want to use private transportation, they charge different fees, it depends on the driver so we don’t know what to expect.” (20-35-year-old women outside the ITS, Bar Elias*)

Although doctors’ fee at PHC facilities is generally lower than the fee at private clinics, some women reported a recent increase in the doctors’ fee at some PHC facilities, which seemed to discourage them from seeking care whenever needed.

*“The fee is still the same at private clinics but it increased at some of the PHC centres.” (15-19-year-old women outside the ITS, Bar Elias)*

The costs of obtaining a modern contraception method also seemed to have increased at some health facilities since the crisis. Many women reported their inability to have an IUD inserted because of the recent increase in its costs. Also, some women reported that many doctors stopped inserting IUDs at PHC facilities, which used to cost 3000 LBP ($2 before inflation) and did it instead at their private clinics, which incurred additional costs.

*“I went to a health centre where they are supposed to be provided for free or at least cheap, I could not have an IUD inserted because the nurse told me it is cleaner to have it inserted at a private clinic (with a wink) but it would have cost me 50000 LBP to obtain it at a private clinic so I could not do it.” (Mothers and mothers-in-law outside the ITS, Bar Elias)*

Although many PHC maintained their provision of free modern contraception methods, women were not able to obtain them unless they pay 3000 LBP to cover the doctor’s fee, but women who were struggling financially, were not able to pay the fee.

*“Sometimes they don’t give you the medications unless you go in and pay the doctor’s fee.” (20-35-year-old women outside the ITS, Saad Nayel)*

Although the majority of women reported a recent increase in the doctors’ fees at PHC facilities, a few women reported that there was no such increase at the PHC they visit, which confirms that some PHC maintained their pre-crises provision of FP services and modern contraception. However, it appears that many women were unaware of them. For instance, during one of the FGDs with 20–35-year-old women inside the ITS in Saad Nayel, a few women complained about the recent increase in the doctors’ fee at the health factifies they visited, one woman disagreed saying *“At my centre they still ask for 3000 LBP.”*

Similarly, 15–19-year old-girls in Bar Elias reported no changes in the doctors’ fee at the PHC facilities they visit.

*“The doctor’s fee is still the same but there are more precautions.”(15-19-year-old girls inside the ITS, Bar Elias)*

There were also mixed reviews about the increase in the price of contraception pills at pharmacies. Only women living inside the settlements in Bar Elias reported that they used to buy contraception pills from Syria before the crisis, which is no longer possible due to the closures of borders to control Covid-19 transmission.

*“We used to get them from Syria because they are cheaper but since the borders closed after corona we are obliged to buy them from the pharmacy in Lebanon.” (Mothers and mother- in-law inside the ITS, Saad Nayel)*

Women who were struggling financially decided not to buy modern contraception methods because of other priorities. Consequently, such women reported depending on natural contraception methods.

*“There are people who are struggling to provide their basic needs so they are unable to afford the pills and the IUD. I used to take pills before the crisis but now I am depending on natural methods in agreement with my husband.”(Mothers and mothers-in-law outside the ITS, Bar Elias)*

#### Concern over being infected with Covid-19

Some participants reported their concern about being infected with Covid-19 at the health facilities or on the way to reach the facilities, especially when they are unable to afford face masks, or when they are obliged to use overcrowded public transportation means, which were among the reasons that discouraged them to seek care.

*“Yes definitely, concern over corona is a main factor. Also, we cannot afford to buy masks.” (15-19-year-old girls inside the ITS, Saad Nayel)*

*“We also worry about getting infected with Corona, you feel that everyone around you is carrying the virus.” (20-35-year-old women outside the ITS, Bar Elias)*

#### Road closures and concern over insecurity

The political crisis in Lebanon as well as the pandemic resulted in road closures and insecurity in many locations. The issue of road closures was brought up especially by women who lived outside the ITS and who discussed that road closures and insecurity were among the reasons that discouraged them from seeking health care.

*“The most important thing is the road, the health centre is far. There have been robberies since the demonstrations started, it is not safe anymore.“ (20-35-year-old women outside the ITS, Saad Nayel)*

*“The roads are not the same as before, we used to move freely, it used to be safe. Not anymore. We worry that people may stop us and we also worry about the road closures.” (20-35-year-old women outside the ITS, Bar Elias*)

Refugees living inside the ITS seemed to face additional restrictions as they were not allowed to leave the settlements during the imposed lockdown periods.

## Discussion

This study found that the combined crises in Lebanon affected women’s reported access to FP services and modern contraception. The pandemic and its implications caused an interruption/or change in the provision of FP services and modern contraception. However, the combined effect of the crises may have decreased women’s demand for those services due to additional problems with geographical and financial accessibility and acceptability (Fig. 3) that emerged after the crises. Therefore, it may be worth exploring new strategies to deliver FP and modern contraception methods.

Availability of FP services, including provision of modern contraception, was affected by the crises. On the supply side, there has been a disruption in the provision of modern contraception at PHC facilities causing a reduced supply of pills. Moreover, women reported that there was a disruption in outreach RH services that provide modern contraception. Furthermore, we anticipate that the port explosion in Beirut had an indirect effect on the study population living in Bekaa, as UNHCR and many NGOs redirected their resources and attention towards those directly affected by the explosion in Beirut [33], which may have affected the provision of RH services to refugees residing in Bekaa. On the demand side, many women were unaware about the recent changes in FP service provision since the crises, which may have discouraged them from seeking care.

The disruption in outreach mobile RH services (provided by some NGOs and UN agencies) will likely interrupt care for the most vulnerable women living inside the ITS or in remote locations as they are the least likely to be able to access alternative resources for health care [32]. In Lebanon, the displaced persons and migrant workers who likely suffered most from this interruption, constitute around 30% of the population [34].

The disruption in the contraceptives supply chain caused by the pandemic resulted in shortages of contraceptive products worldwide [35], however, the situation in Lebanon was likely to have been more severe due to the other challenges that the country was facing because of the financial and political crises. Importing medications and medical equipment became increasingly difficult as the LBP lost approximately 80% of its value [36], which likely affected the importation of modern contraception and local supply.

Geographical and financial accessibility to FP services were disrupted as a result of the crises. Because of the risk implications arising from mobility restrictions, road closures and using overcrowded public transportation, and the financial implications arising from using private taxis, many women reported that they did not seek care. Moreover, the reduced supply of contraception pills obliged women to return each month to obtain an additional supply, which had additional financial implications. Similarly, women who wanted to acquire IUDs at PHC facilities were often asked to get them at private clinics for a much higher fee, which was not the case before the crises. As a result, many women may have prioritized feeding their families over seeking costly FP services or using modern contraception.

A recent scoping review on the impact of Covid-19 pandemic on access and utilization of SRH services described that barriers to accessing such services globally included transportation disruptions, financial difficulties and reduced medical supplies and human resources and highlighted that these barriers were more prominent in countries with economic disadvantages [37], which is the case in Lebanon.

Syrian refugee women reported their dissatisfaction with using PHC for RH before the crises. However, it seems that their acceptability of FP services was exacerbated since the crises. The main reported issues regarding provision of FP services and modern contraception that discouraged women from seeking care as described by participants were Covid-19 regulations imposed at PHC facilities as well as maltreatment by staff at those facilities. On the supply side, new Covid-19 regulations, which obliged some women to wait for long hours in the sun, as well as the increase in reported maltreatment from health providers, which may be related to proper implementation of Covid-19 policies, could have influenced refugee women’s acceptability of provided FP services and therefore decreased their demand for them.

On the demand side, issues related to women’s unawareness about the change in service provision including new Covid-19 regulations at PHC facilities may have contributed to reducing their acceptability of such services. A few women indicated that some health facilities maintained their pre-crises provision of FP services and modern contraception methods, however, many women did not know about them. Since PHC facilities operating under the MoPH are unlikely to have different regulations regarding doctors’ fees, it is unlikely that doctors’ fees increased at some of those facilities but not others. It seems that refugees that reported such an increase either attended care at PHC that were not supported by UNHCR, they were no longer supported by UNHCR themselves, or they attended care at other types of health facilities that were not operating under the MoPH. Either way, it is clear that many women lacked adequate information about provision of FP services and modern contraception to Syrian refugees.

Some possible ways to address these barriers include sensitizing refugee women about transmissible diseases including Covid-19 and its dangers as well as the updated regulations at PHC facilities that are still being applied. This could increase the likelihood of women respecting those regulations, decrease the possibility of being maltreated by staff at PHC facilities, and is likely to increase women’s acceptability of FP services and eventually their demand for them.

To address some of the reported barriers to accessing and using FP and modern contraception during the pandemic and even after the pandemic, mobile health (mHealth), which involves the use of mobile technologies and multimedia tools to achieve health goals and support healthcare delivery [38, 39], can be used to deliver information and consultations to refugee women [40]. This could include sensitizing refugee women about transmissible diseases including Covid-19 and its dangers, delivering Information on changes in service provision during the pandemic and on available modern contraception methods for lower prices and in locations that are closer to where women live. Many women reported being mistreated at PHC facilities. While this could have happened at some facilities, it was probably triggered by refugee women’s lack of knowledge about PHC facilities that are supported by UNHCR and also not knowing if they are entitled to receive such support, as only women who are registered with UNHCR can receive it. Using mHealth to inform refugee women about the PHC that are supported by UNHCR as well as the type of support they are entitled to receive may increase their acceptability of services provided at those facilities. Information can be provided through platforms such as WhatsApp, which is widely used among Syrian refugees in Lebanon [40]. The use of text messages and telephone reminders have been successful in collecting health‐related information from pregnant women and in increasing access to FP information in low-income settings [41, 42].

To address the issue of long waiting times and having to interact with staff at PHC facilities, mHealth can be used to provide consultation for contraceptives where women only attend the health facility to pick up the medications. The use of mHealth technology can also help women overcome issues related to provider prejudice, stigmatization, discrimination and lack of privacy and confidentiality [43]. However, programs intended to use such technology should take into account the low literacy levels among refugees, the network coverage in areas where refugees live, and the fact that a phone may be shared by family members [40]. In that case, it may be best if such services were coupled with mobile outreach RH services, especially for women living inside the ITS with limited mobility [44]. Expanding the use of mobile outreach services can also address issues with geographical and financial accessibility to FP services or when commuting is not secure.

One potential solution to address the disruption of contraception supply is to consider providing Long Acting Reversible Contraception (LARC) such as subdermal implants that were provided by some NGOs before the crises. Refugee women need to be educated about the different types of LARC to ensure that they are making an informed choice and selecting the method that best meets their personal, reproductive and health needs [45]. Making an informed choice when selecting one of the LARC methods may address the problem of early discontinuation associated with their use in some low-income settings [46, 47]. Using mHealth technology can be used to educate women about the different types of LARC methods and their side effects and to inform them about health facilities that have qualified personnel and which provide such methods for free or for a subsidized fee.

Providing such contraceptives was recommended by the International Federation of Gynaecology and Obstetrics’ Contraception and FP Committee in the early days of the pandemic [48]. LARC have low failure rate and women do not have to come back for constant resupply as with contraception pills [49]. Moreover, it seems that many women favoured using them even before the pandemic given the inconvenience of using pills for highly mobile refugee women living in poor conditions [19].

A recent systematic review indicated that training healthcare providers in how to administer LARC may increase the uptake among women in low and middle-income countries (LMIC) [50]. Moreover, A recent review showed that LARC are more cost effective than short acting reversible contraceptive methods, especially after one year of use, however, only 2 of the included studies in this review were from LMIC [51].

### Strengths and limitations

What makes this study different from other studies that explore the effects of Covid-19 on FP services is that the disruption of access to those services in Lebanon was amplified due to the overlapping crises. The overlapping crises are expected to extend beyond the pandemic. Therefore, it is essential to discuss potential solutions to increase access to RH services including FP given those crises, which may be useful in other similar settings.

This is a qualitative study and does not aim to be representative of all Syrian refugee women in Lebanon but rather to understand the current provision of FP services and modern contraception and health seeking behaviour during the crises. The interactions that take place within a FGD may have resulted in a few participants dominating the conversation, may have discouraged some participants from discussing controversial perspectives, or may have encouraged tendencies towards normative discourses [52]. We tried to avoid these issues by encouraging participants to express themselves and by using additional probing strategies.

Interviewing health providers and health officials would have enriched our data on provision of FP services and modern contraception but this was difficult to arrange due to the pandemic and the 4^th^ of August explosion.

Due to the pandemic, it was impossible to conduct face-to-face interviews, the gold standard in qualitative research. However, the use of video calls, increasingly used in recent years and the closest method that creates an in-person experience whilst geographically separate, proved to be as effective [53]. Therefore, the use of video calls is well justified given the pandemic constraints.

## Conclusion

The overlapping crises in Lebanon affected Syrian refugee women’s access to FP services and modern contraception by affecting their availability, geographical and financial accessibility and acceptability, which made exploring new strategies to deliver FP services and modern contraception extremely important. We discussed a number of potential solutions that seem promising in other low-income settings such as using mHealth technology [41, 42, 54], using outreach mobile services to provide FP and modern contraception [55] and providing LARC [49], which could address several barriers simultaneously and which could be more cost effective on the long run. Even though the pandemic has receded, the political and financial crises are likely to persist for some time in Lebanon. Therefore, future research should focus on exploring the long-term feasibility, acceptability and cost effectiveness of implementing some of the discussed solutions in Lebanon.

### Supplementary Information

**Additional file 1. **Conceptual framework

## Data Availability

Data from FGDs is available in the Population Council Dataverse (Covid-19 Research and Evaluations) and can be accessed via the following link: https://doi.org/10.7910/DVN/A44ZWF

## Abbreviations

FGDs: Focus Group Discussions
FP: Family Planning
ITS: Informal Tented Settlements
IUD: Intrauterine Device
LARC: Long Acting Reversible Contraceptives
LBP: Lebanese Pound
LMIC: Low and Middle-Income Countries
MoPH: Ministry of Public Health
NGOs: Non-Governmental Organizations
PHC: Primary Health Care
RH: Reproductive Health
SRH: Sexual and Reproductive Health
UN: United Nations
UNHCR: The United Nations High Commissioner for Refugees
