# Supplementary material for: Syrian refugee women’s access to family planning services and modern contraception during overlapping crises in Bekaa, Lebanon
Source: BMC Womens Health. 2023 Sep 6;23:475. doi: 10.1186/s12905-023-02613-8 (PMC10481481; doi:10.1186/s12905-023-02613-8)
Supplement: Supplementary file 1 — Additional file 1. Conceptual framework [file 12905_2023_2613_MOESM1_ESM.docx]

Additional file 1: Conceptual framework

|  | **Supply side barriers** | **Demand side barriers** |
| --- | --- | --- |
| **Availability** | Availability of RH services and modern contraception methods | Information on available services and contraception methods |
| **Geographical accessibility** | Service location, mobility restrictions | Household location, availability of transportation |
| **Financial accessibility** | Cost of services, medications and transportation | Household resources and willingness to pay |
| **Acceptability** | Characteristics of the health service including rules and regulations | Women’s attitudes and expectations |
